# 

**Published:** 1886-04

**Authors:** 


					﻿MOLLERS w’ecTah
COD-LIVER 0l|
PUREST
BEST.
FOR
General
Debility,
Scrofula,
Rheumatism
or Consumption.
Is superior to any in de-
licacy of taste and smell,
medicinal virtues and purity.
London, European and New
York physicians pronounce it the
I purest and best. Sold by Druggists.
W.H.Schieffelin & Co.(K^S) NewYork
				

